# EMA - A R package for Easy Microarray data analysis

**DOI:** 10.1186/1756-0500-3-277

**Published:** 2010-11-03

**Authors:** Nicolas Servant, Eleonore Gravier, Pierre Gestraud, Cecile Laurent, Caroline Paccard, Anne Biton, Isabel Brito, Jonas Mandel, Bernard Asselain, Emmanuel Barillot, Philippe Hupé

**Affiliations:** 1 Institut Curie, Paris F-75248, France; 2 INSERM, U900, Paris F-75248, France; 3 Ecole des Mines ParisTech, Fontainebleau, F-77300 France; 4 Institut Curie, Departement de Transfert, Paris F-75248, France; 5 CNRS, UMR144, Paris F-75248, France; 6 CNRS, UMR3347, Orsay F-91405, France; 7 INSERM, U1021, Orsay F-91405, France; 8 Université Paris-Sud 11, Orsay F-91405, France

## Abstract

**Background:**

The increasing number of methodologies and tools currently available to analyse gene expression microarray data can be confusing for non specialist users.

**Findings:**

Based on the experience of biostatisticians of Institut Curie, we propose both a clear analysis strategy and a selection of tools to investigate microarray gene expression data. The most usual and relevant existing R functions were discussed, validated and gathered in an easy-to-use R package (EMA) devoted to gene expression microarray analysis. These functions were improved for ease of use, enhanced visualisation and better interpretation of results.

**Conclusions:**

Strategy and tools proposed in the EMA R package could provide a useful starting point for many microarrays users. EMA is part of Comprehensive R Archive Network and is freely available at http://bioinfo.curie.fr/projects/ema/.

## Findings

Numerous analysis methods and tools have been developed to study microarray, many of them being implemented as free R [1] and/or Bioconductor [2] packages. This abundance of methods makes choosing the best approach difficult for newcomers and non-specialist users.

Based on the experience of the biostatisticians of Institut Curie, we propose a clear analysis strategy combining a large variety of standard methodologies. The most usual and relevant R functions needed to perform these analyses were selected and gathered in the R package EMA (Easy Microarray data Analysis). EMA covers an entire analysis process including quality control, normalisation, exploratory analysis, unsupervised and supervised classification, functional analysis and censored data exploration. The package can be used for both one or two-colours gene expression micrarrays and for exon expression experiments.

### Analysis strategy

Firstly, the quality of the data must be assessed in order to detect problematic raw probe-level data, such as spatial artifacts on the chip or poor quality hybridisation. Indeed, gene expression experiments suffer from many sources of technical and experimental variation. Removing noise and systematic biases is performed in order to both improve the biological signal and make all the arrays comparable. This is the so-called normalisation step. Secondly, we propose to discard the probesets with very low signal across the samples (*i.e*. genes unexpressed or below detection threshold). This filtering step leads to both a noise reduction in the data and an increase in the statistical power of the subsequent analysis.

Then, exploratory approaches are classically used to find clusters of genes (or samples) with similar profiles. Note that here, biological interpretation depends on the choice of the similarity metrics. These approaches potentially highlight outliers and/or non relevant effects (batch effect for example), which can be subsequently estimated and/or removed from the data thanks to appropriate methods.

Finally, supervised approaches aim at the identification of differentially expressed genes (DEG), or deregulated pathways by taking into account the multiple testing issues. The biological interpretation of the differential analysis results can be performed thanks to functional and gene set enrichment analyses. Sample class prediction (eg good vs poor clinical outcome) based on supervised classification methods can also be performed to highlight genes signatures.

### Selected tools

**Figure 1:**
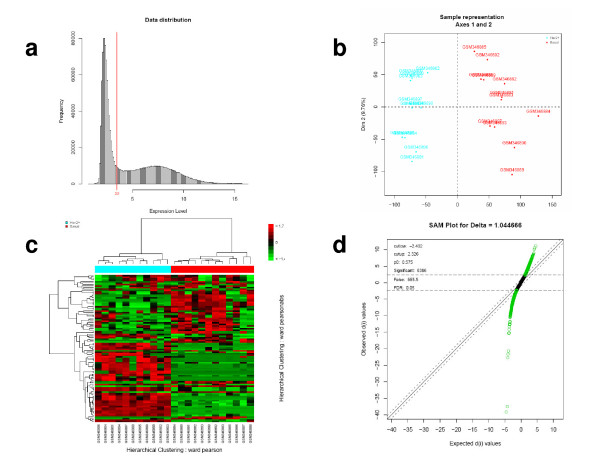
**Graphical outputs provided by the EMA package for the class comparison study of **[18]. (a) Histogram of probesets expression values across the 23 samples after GCRMA normalisation and log2 transformation. Probesets with an expression value below 3.5 (red vertical line) are discarded. (b) Individuals factor map produced by the PCA performed on the 23 filtered gene expression profiles. (c) Heatmap of the 23 gene expression profiles based on the 100 genes with the highest interquartile range (IQR) values. Sample clustering was performed using Pearson's correlation coefficient and Ward criterion. Gene clustering was performed using absolute Pearson's correlation coefficient and Ward criterion. (d) Qqplot produced by the SAM analysis on the two groups of tumours. Probesets in green are considered to be differentially expressed between the two conditions.

For the data quality assessment, we recommend to use the arrayQualityMetrics package [3], which performs a powerful, easy-to-use and comprehensive data quality estimation as well as an automatic html report. The EMA package proposes the most famous techniques for Affymetrix GeneChip normalisation: MAS5.0 [4], RMA [5] and GCRMA [6]. We recommend to use GCRMA because it outperforms the other approaches (by ignoring the mismatch intensities and taking into account the probe sequence information) and allows an efficient filtering of irrelevant probesets thanks to its bimodal distribution of probesets expression values (Figure 1a). Other packages such as limma [7], vsn [8] or lumi [9] can be used to normalise non Affymetrix data. After this first step, the main EMA functions can be used for any type of expression data, using a simple data expression matrix as input.

The EMA package provides functions to perform exploratory analyses such as Principal Component Analysis (PCA, Figure 1b), hierarchical clustering (Figure 1c) or Multiple Factor Analysis. They are based on R packages such as FactoMineR [10], cluster [11], or mostclust [12]. The use of linear model is proposed to estimate and to remove the non relevant effects potentially detected.

Various methods are proposed to perform differential analysis and their choice depends on the sample size. The multtest package provides standard approaches like Student or Mann-Whitney test associated with multiple testing correction methods. The Significance Analysis of Microarrays (SAM) approach [13] (siggenes package) is also very interesting because it both estimates the null distribution and takes into account the correlation between probesets (Figure 1d). The rank product method [14] (RankProd package) dedicated to small sample size dataset is also offered, as well as some linear model (ANOVA) functions. Alternatively, the user can apply the limma package which is a very powerful tool to assess differential expression by linear models.

The functional enrichment of the DEG list is assessed based on the GeneOntology [15], and KEGG [16] pathways annotation terms. The hyper-geometric test of the GOstats package is used to test the over-representation of the functional terms in the gene list.

For sample class prediction, we suggest to use the CMA package [17] including the most popular machine learning and gene selection algorithms. In the context of censored data, the EMA package supports Kaplan Meier and log-rank analyses using the survival package.

### Example

The proposed analysis strategy was applied to the breast cancer gene expression dataset [18] comparing 12 Basal-like carcinomas (BLCs) and 11 HER2 positive carcinomas (HER2+). Some graphical outputs for data preprocessing, exploratory analysis and differential analysis steps are displayed in Figure 1. The RNA profiles were analysed using U133 plus 2.0 Affymetrix GeneChip. Three genes (P-cadherin, v-kit, FOXC1) were reported by the authors to be associated to a genes cluster over-expressed in the basal-like carcinomas and three genes (PTEN, Her2 and GRB7) to a genes cluster over-expressed in the Her2+ carcinomas. All these genes but one (v-kit) were found to be differentially expressed using the EMA package. This discrepancy is easily explained because in spite of v-kit belongs to a basal-like expression cluster, no change in v-kit expression can be observed between the two groups in this clustering analysis. This is because the hierarchical clustering was performed on genes (such as v-kit) not necessary differentially expressed between the two populations.

The R scripts used to analyse this gene expression dataset can be found in [Additional file 1]. Transcriptomic data used in this application are publicly available at Gene Expression Omnibus (Accession number: [GSE13787]) and are part of the package.

## Conclusions

EMA is a freely available R package which implements a complete strategy for expression microarray analysis. The package includes a vignette [Additional file 2] which describes the detailed biological/clinical analysis strategy used at Institut Curie. Most of the functions were improved for ease of use (fewer command lines, default parameters tested and chosen to be optimal). Relevant, enhanced and easy-to-interpret text and graphic outputs are offered. The package is available on The Comprehensive R Archive Network repository [19].

## Availability and requirements

• Project Name: EMA

• Project home page:

http://bioinfo.curie.fr/projects/ema/

http://cran.r-project.org/

• Operating systems: Linux, Windows

• Programming language: R

• Other requirements: R version ≥ 2.10. R packages: cluster, Hmisc, heatmap.plus, FactoMineR, GOstats, survival, multtest, affy, gcrma, rgl, GSA, RankProd, siggenes, MASS, hgu133plus2.db, xtable, biomaRt.

• License: GNU GPL

• Any restrictions to use by non-academics: none

## Competing interests

The authors declare that they have no competing interests.

## Authors' contributions

NS and EG discussed the choice of the strategy and tools, participated to the development of the EMA package and wrote the paper. PG, CL, CP, AB, IB, JM discussed the choice of the strategy and tools and participated to the development of the EMA package. BA, EB and PH discussed the choice of the strategy and tools and supervised the work group. All authors read and approved the final manuscript.

## Supplementary Material

Additional file 1 **R scripts applied to the breast cancer gene expression dataset **[18]. R script used to analyse the breast cancer gene expression data set [18]. Click here for file

Additional file 2 **EMA vignette**. The vignette discuss the detailed biological/clinical analysis strategy used at Institut Curie and presents an application to a gene expression dataset. Click here for file
