# Supplementary material for: EMA - A R package for Easy Microarray data analysis
Source: BMC Res Notes. 2010 Nov 3;3:277. doi: 10.1186/1756-0500-3-277 (PMC2987873; doi:10.1186/1756-0500-3-277)
Supplement: Additional file 1 — R scripts applied to the breast cancer gene expression dataset [18]. R script used to analyse the breast cancer gene expression data set [18]. [file 1756-0500-3-277-S1.DOC]

**Additional File 1: R scripts applied to the breast cancer gene expression dataset (Marty et al, Breast cancer research, 2008)**

##Load EMA package

###########################################################################

require(EMA)

##GCRMA Normalisation and filtering

###########################################################################

##Not run because cel files are not available from this package

cel.path=paste(getwd(),"/Data/E-GEOD-13787", sep="")

marty<-normAffy(cel.path, method="GCRMA")

##Load GCRMA Normalised data

##marty.type.cl=Her2+ corresponds to Her2+ breast cancer

##marty.type.cl=Basal corresponds to Basal-Like carcinoma

data(marty)

##And discard probesets with a maximum log2 expression value below 3.5

marty.f<-expFilter(marty)

dim(marty.f)

##Principal Component Analysis

###########################################################################

acp<-runPCA(t(marty.f), scale=FALSE,lab.sample=marty.type.cl, plotSample=FALSE, plotInertia=FALSE)

plotInertia(acp)

## Individual map (axe 1 and 2)

plotSample(acp,axes=c(1,2),lab=marty.type.cl)

## Or create a pdf report with selected plots

runPCA(t(marty.f), scale=FALSE, pdfname="PCA.pdf", lab.sample=marty.type.cl)

## PCA after normalisation and without filtering, but with scaling

acp<-runPCA(t(marty), scale=TRUE, lab.sample=marty.type.cl, plotSample=FALSE, plotInertia=FALSE)

## Individual map (axe 1 and 2)

plotSample(acp,axes=c(1,2),lab=marty.type.cl)

##Gene representation (only genes the most correlated to the two first components)

level.cl<-ifelse(apply(marty,1,max)>3.5,"High","Low")

plotVariable(acp,axes=c(1,2),lim.cos2.var=0.8,lab=level.cl,label="")

##Agglomerative hierarchical clustering

###########################################################################

## Sample Hierarchical Clustering (Pearson's correlation coefficient and Ward method)

c.sample<-clustering(data=marty.f, metric="pearson", method="ward")

clustering.plot(tree=c.sample, lab=marty.type.cl, title="GCRMA Data - filtered")

## Heatmap performed on the 100 probesets with the highest IQR values

mvgenes<-genes.selection(marty.f, thres.num=100)

c.sample<-clustering(data=marty.f[mvgenes,], metric="pearson", method="ward")

c.gene<-clustering(data=t(marty.f[mvgenes,]), metric="pearsonabs", method="ward")

clustering.plot(tree=c.sample, tree.sup=c.gene, data=marty.f[mvgenes,], names.sup=FALSE, lab= marty.type.cl, trim.heatmap=0.99)

##Differential analysis

###########################################################################

###Student test with BH correction and qqplot of genes.

marty.type.num <- ifelse(marty.type.cl=="Her2+",0,1)

rt<-runTtest(marty.f, labels=marty.type.num,algo="t.equalvar", q=0.05)

head(rt)

###SAM Analysis

rs<-runSAM(marty.f, labels=marty.type.num)

head(rs)

##Gene Annotation

###########################################################################

##Sort by SAM significance

rs.sorted <- rs[order(abs(rs$Stat), decreasing=TRUE),]

rs.sign <- rs.sorted[which(rs.sorted[,"Significant"]),]

rs.annot<-bioMartAnnot(rs.sign, inputTypeId ="affy_hg_u133_plus_2", outputTypeId = c("entrezgene","hgnc_symbol"), dataset=c("hsapiens_gene_ensembl"), database = "ensembl")

head(rs.annot)

##check the signal of the 11 probes summarized in each probeset.

## Not run because cel files are not available from this package

filenames <- list.files("Data/E-GEOD-13787", pattern=".CEL", ignore.case=TRUE)

rawdata <- ReadAffy(filenames=filenames, celfile.path="Data/E-GEOD-13787", cdfname=NULL)

probePlots(rawdata, pbsList=rs.annot$affy_hg_u133_plus2[1:10])

##GeneSet Analysis

###########################################################################

## http://www.broad.mit.edu/gsea/msigdb/msigdb_index.html

## You have to register first and then download the gmt file from their site

gsaOUT <- runGSA(marty.f, marty.type.num , gmtfile="c2.kegg.v2.5.symbols.gmt", chip="hgu133plus2")

##Functional enrichment assessment

###########################################################################

## GO and KEGG analysis on the DEG by the SAM procedure

runHyperGO(list=rownames(rs.sign), pack.annot="hgu133plus2.db", name="HyperGO_type")

runHyperKEGG(list=rownames(rs.sign), pack.annot="hgu133plus2.db", name="HyperKEGG_type")

##Survival Analysis

###########################################################################

set.seed(5000)

gene<-rnorm(100)

gene[51:100]<-gene[51:100]+2

group<-ifelse(gene<=median(gene),"Low gene expression","High gene expression")

time<-abs(rnorm(100))

time[51:100]<-time[51:100]+2

status<-sample(c(0,1),size=100,replace=TRUE)

res<-km(time,status,group,title="Kaplan Meier curve")

res$fit.km

res$lr

res$p.lr
