# Supplementary material for: EMA - A R package for Easy Microarray data analysis
Source: BMC Res Notes. 2010 Nov 3;3:277. doi: 10.1186/1756-0500-3-277 (PMC2987873; doi:10.1186/1756-0500-3-277)
Supplement: Additional file 2 — EMA vignette. The vignette discuss the detailed biological/clinical analysis strategy used at Institut Curie and presents an application to a gene expression dataset. [file 1756-0500-3-277-S2.PDF]

EMA - Easy Microarray data Analysis

N. Servant [1,2,3], E. Gravier [1,2,3,4], P. Gestraud [1,2,3], C. Laurent [1,2,3,6], C. Paccard [1,2,3],
A. Biton [1,2,3,5], I. Brito [1,2,3], J. Mandel [1,2,3], B. Asselain [1,2,3], E. Barillot [1,2,3], P. Hupé [1,2,3,5].
[1]Institut Curie, Paris F-75248, France,
[2]INSERM U900, Paris F-75248, France,
[3]Ecole des Mines ParisTech, Fontainebleau, F-77300 France,
[4]Institut Curie, Departement de Transfert, Paris F-75248, France,
[5]CNRS, UMR144, Paris F-75248, France
[6]CNRS, UMR146, Paris F-75248, France

ema-package@curie.fr - http://bioinfo.curie.fr/projects/ema/

October 27, 2010

Contents

1 Introduction **2**

2 Data Input **2**

3 Quality Control **2**

4 Data pre-processing **3**
4.1 Normalisation 3
4.2 Data filtering 4

5 Exploratory Analysis **5**
5.1 Principal Component Analysis 5
5.2 Agglomerative Hierarchical Clustering 8

6 Differential Analysis **9**

7 Gene annotation **11**

8 Gene-Set analysis **12**

9 Functional enrichment assessment **13**

10 Supervised Classification **13**

11 Survival analysis **14**

12 System Performance **15**

13 Session Info **16**

1 Introduction

The increasing number of methodologies and tools currently available to analyse gene expression microarray data can be confusing for non specialist users. Based on the experience of biostatisticians of Institut Curie, we propose both a clear analysis protocol and the tools to investigate the data. It provides a useful starting point for many microarrays users.
All the functions included in the powerful and free EMA (Easy Microarray data Analysis) package were tested and chosen according to their performance and their suitability.
Most of these functions have also been improved to facilitate their use, the visualization and the interpretation of the results in the specific field of microarray data analysis.
EMA supports entire analysis process for any type of gene expression arrays, from normalisation, unsupervised and supervised analysis to functional analysis. Exploratory analyses are also proposed in the context of censored data.
Below, we present the analysis strategy used in the Institut Curie for a class comparison study. This strategy is applied to a cancer microarrays dataset (Marty *et al.* (2008) - GSE13787) which compares 12 Basal-like breast carcinomas (BLCs) and 11 HER2 positive breast carcinomas (HER2+). The mRNA profiles of these 23 samples were analysed using HG-U133plus2 Affymetrix chips that measure the expression values of 54613 probesets (after removal of AFFX probesets).
The aim is to detect differentially expressed genes between the two groups of tumours, in order to discover potential therapeutic targets in human BLCs. A brief application to simulated survival data is also provided.

2 Data Input

The main functions of the EMA package work with an expression matrix as input (expression levels -from e.g genes- measured in tissue samples and log2 transformed). Based on this simple input, the EMA package can be used for any gene expression data such as Affymetrix data (HG-U133a/b, HG-U95av2, HG-U95a/b/c/d/e, HG-U133plus2, MouseGenome4302/430a2, MurineGenome11k/19k), one-colour experiments like Illumina data (HumanWGv1/2/3, MouseWG1/2, RatRSv1), two-colour data or exon data (HuEx1.0stv2). The annotation data must be available from the BioConductor project (Gentleman *et al.* (2004)) or the BrainArray website (Dai *et al.* (2005)) to work with data annotation and to perform the functional analysis. Some of the EMA functions can also be used for other applications like miRNAs expression data. The only limiting step which depends on the providers and the data type is the normalisation (see section 4.1).

3 Quality Control

Data quality control is a major concern in microarray analysis. This step aims to detect problematic raw probe-level data (array with spatial artifacts or with poor RNA quality for example) to facilitate the decision of whether to remove this array from further analysis. However, raw probe-level data artefacts can also be removed thanks to normalisation procedures (see section 4.1).
This is why we recommend to combine the results obtained by both the quality control step and the normalisation process to make a decision. For quality assessment, we recommend to use the arrayQualityMetrics package (Kauflmann *et al.* (2009)) which proposes powerful and comprehensive tools and an automatic report generation. This package can be used for Affymetrix datasets as well as for two-colour or non Affymetrix one colour experiments.

4 Data pre-processing

4.1 Normalisation

Microarray normalisation is a fundamental step which aims at removing systematic bias and noise variability caused by technical and experimental artefacts.
This is also the only step which depends on the provider and the data type. For each type of arrays, the raw data files are different and need a specific pre-processing.
Several packages already exist to normalise Illumina, one or two-colour data.
The lumi package (Du *et al.* (2008)) supports the Illumina raw data output of the Illumina Bead Studio toolkit and can easily normalise them.

```
> ## ILLUMINA CHIPS
> ## From lumi1.14.0 help page
> ## load example data
> require(lumi)
> data(example.lumi)
> ## Do all the default preprocessing in one step
> lumi.N <- lumiExpresso(example.lumi)
> ## Convert data into matrix for EMA
> lumi.mat <- exprs(lumi.N)
```

The vsn (Huber *et al.* (2002)) package can be used to normalise data from spotted two-colour or one-colour microarrays as described in the following example. It also includes normalisation techniques for Affymetrix arrays.
Other packages like limma Smith *et al.* (2003) or more recently BABAR (Alston *et al.* (2010)) also provide several normalisation techniques for these data.

```
> ## cDNA CHIPS
> ## From vsn3.16.0 help page
> ## VSN normalisation
> require(vsn)
> data(lymphoma)
> lym <- justvsn(lymphoma)
> ## Convert data into matrix for EMA
> lym.mat <- exprs(lym)
```

EMA offers a function to normalise the Affymetrix GeneChip data using MAS5.0 (Affymetrix Inc. (2002)), RMA (Irizarry *et al.* (2003)) or GCRMA (Wu *et al.* (2004)) methods.
We recommend the use of GCRMA method because it outperforms MAS5.0 by ignoring the MM intensities (observed to detect some specific signal) and it improves RMA by taking into account the sequence information to describe background noise. However, GCRMA can be more time and memory consuming for a large number of arrays. In that case, RMA could be an interesting alternative. The performances of the GCRMA method are shown in the "System performance" section.

```
> ##Load EMA package
> require(EMA)
> ## GCRMA Normalisation
> ## Not run because cel files are not available from this package
> cel.path=paste(getwd(),"/Data/E-GEOD-13787", sep="")
> marty<-normAffy(celfile.path=cel.path, method="GCRMA")
```

4.2 Data filtering

The aim of the filtering step is to discard the probesets with very low expression across the samples (and that provide no biological information) in order to reduce noise in data and to avoid wrong interpretations of the final results.
In differential analysis for example, the number of hypothesis to test is therefore reduced (which is very useful in the multiple testing context).
Another problem also occurs in case of data scaling (for example in PCA) where the biological signal is completely shrinked by the probesets with very low variability.
Most of the probesets with a low variability have either a very low expression level or, on the contrary, have reached their saturation level. However, as shown in the following histogram, those with a low expression level are much more frequent. That's why by discarding the probesets with a low expression level for all the samples, and with a low variability, we expect to easily reduce the problem of noise and data scaling. Other common filtering methods include filtering by variance, inter-quartile range (IQR) or MAS detection call (for Affymetrix arrays).

```
> ##Load GCRMA Normalised data
> ##marty.type.cl=Her2+ corresponds to Her2+ breast cancer
> ##marty.type.cl=Basal corresponds to Basal-Like carcinoma
> data(marty)
> ##And discard probesets with a maximum log2 expression value below 3.5
> marty.f<-expFilter(marty)
> dim(marty.f)
```

```
[1] 33409    23
```

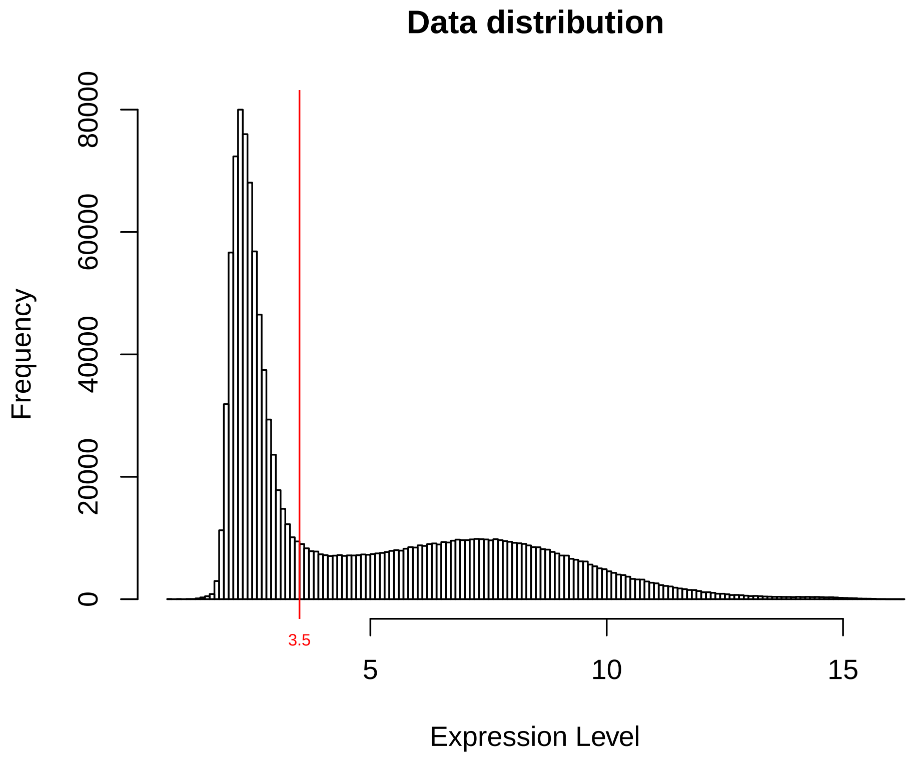

By displaying a bimodal distribution, this histogram supports the choice to use the threshold value of 3.5 that divides low and high expression values.

5 Exploratory Analysis

The aim of exploratory analysis is to explore the gene expression data structure. First, it is recommended to look at the distribution of arrays and/or genes to give a simple summary of the data structure. Among exploratory techniques, PCA or clustering are widely used as they provide easy-to-interpret graphical representations. They make it possible to detect potential outliers and/or bias like batch effect or any non relevant effects. In addition, clusters of similar gene profiles and similar sample profiles can also be identified.

5.1 Principal Component Analysis

The principal component analysis (PCA) is a standard tool for extracting relevant information from a dataset. The goal is to reduce the dataset to a lower dimension and to reveal the underlying structure of the data. As mentioned in the filtering step, scaling probesets with very low variability can introduce a bias in the results (see below), that is why we recommend to perform PCA after filtering.

```
> acp<-runPCA(t(marty.f), scale=FALSE,lab.sample=marty.type.cl,
+             plotSample=FALSE, plotInertia=FALSE)
> plotInertia(acp)
```

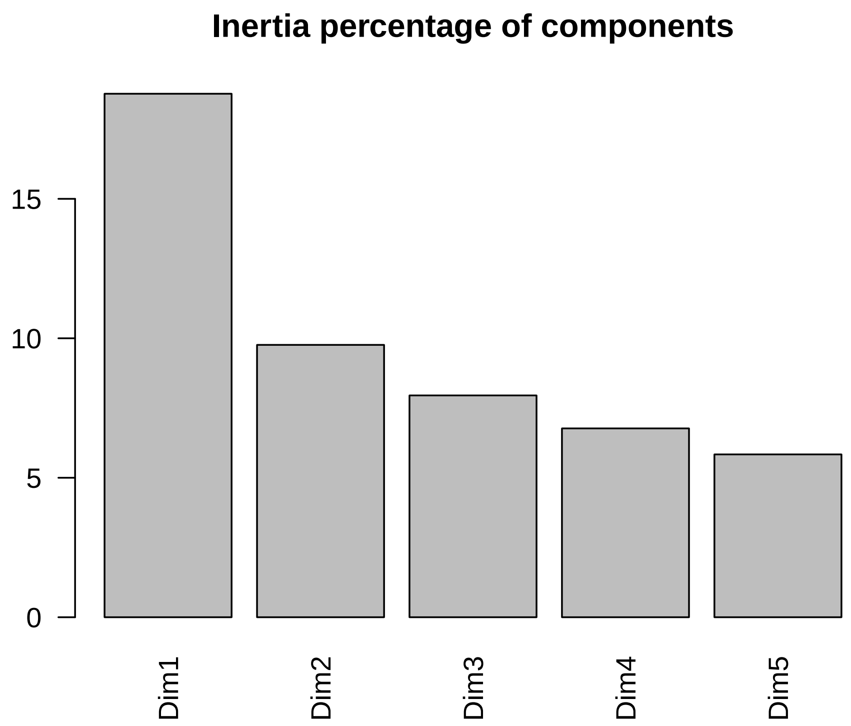


```
> ## Individual map (axe 1 and 2)
> plotSample(acp,axes=c(1,2),lab=marty.type.cl)
```

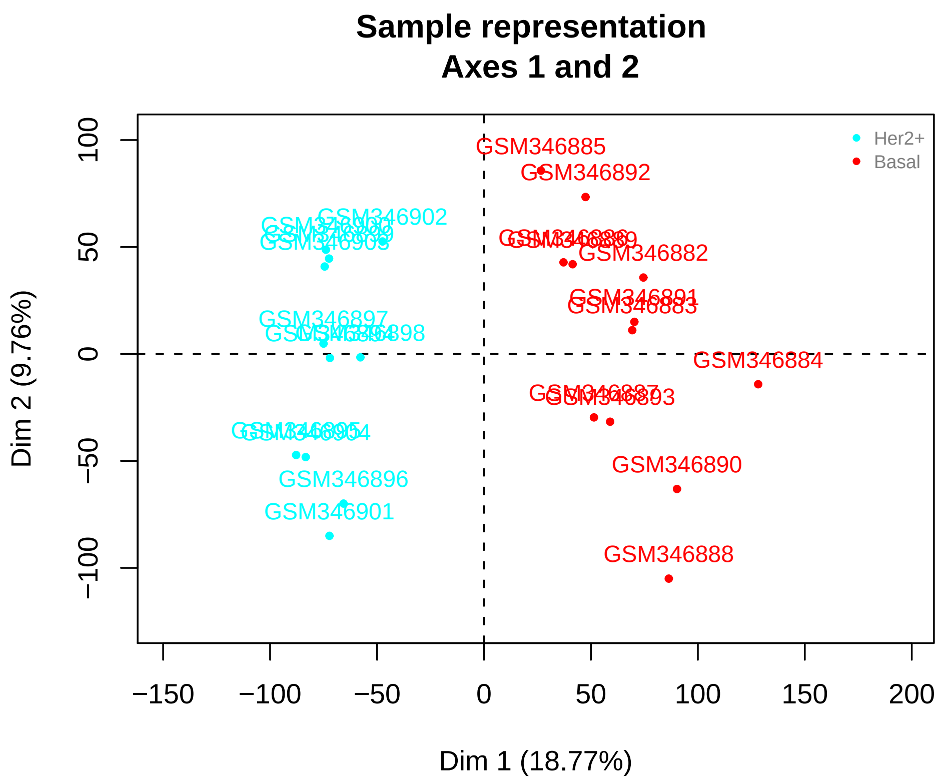


```
> ## Or create a pdf report with selected plots
> runPCA(t(marty.f), scale=FALSE, pdfname="PCA.pdf",lab.sample=marty.type.cl)
```

The two groups of tumours (HER2+ in red and BLCs in blue) are clearly separated by their gene expression profile.

In the following example, we show how the filtering step affects the PCA results. In the case of scaled data, the weights associated to genes with low expression across samples become high and can lead to some wrong interpretations.

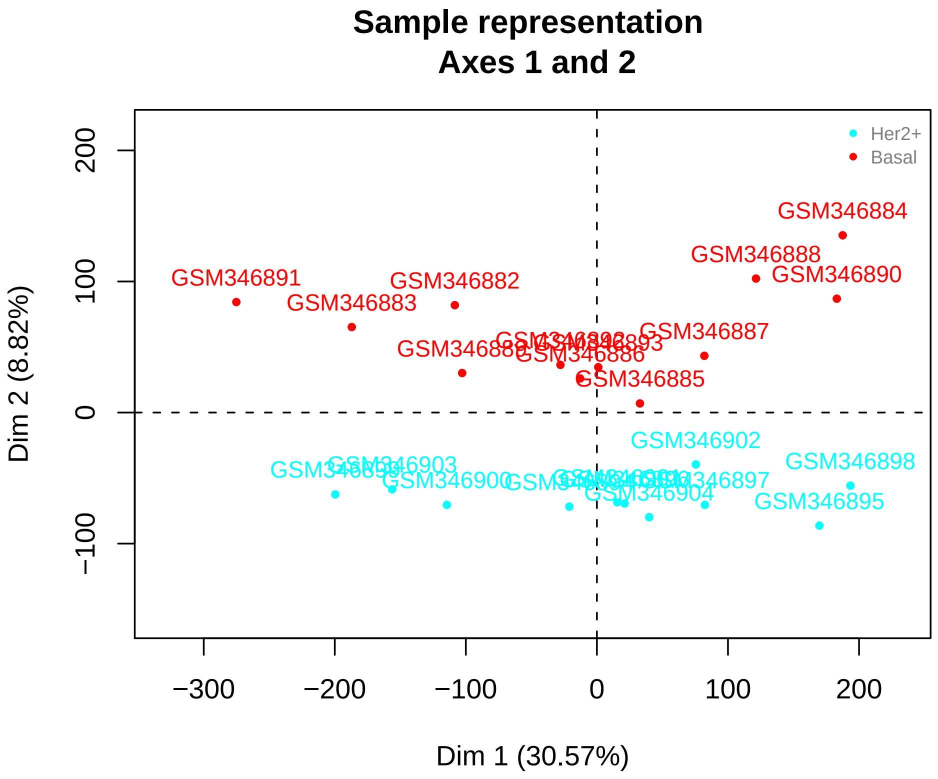

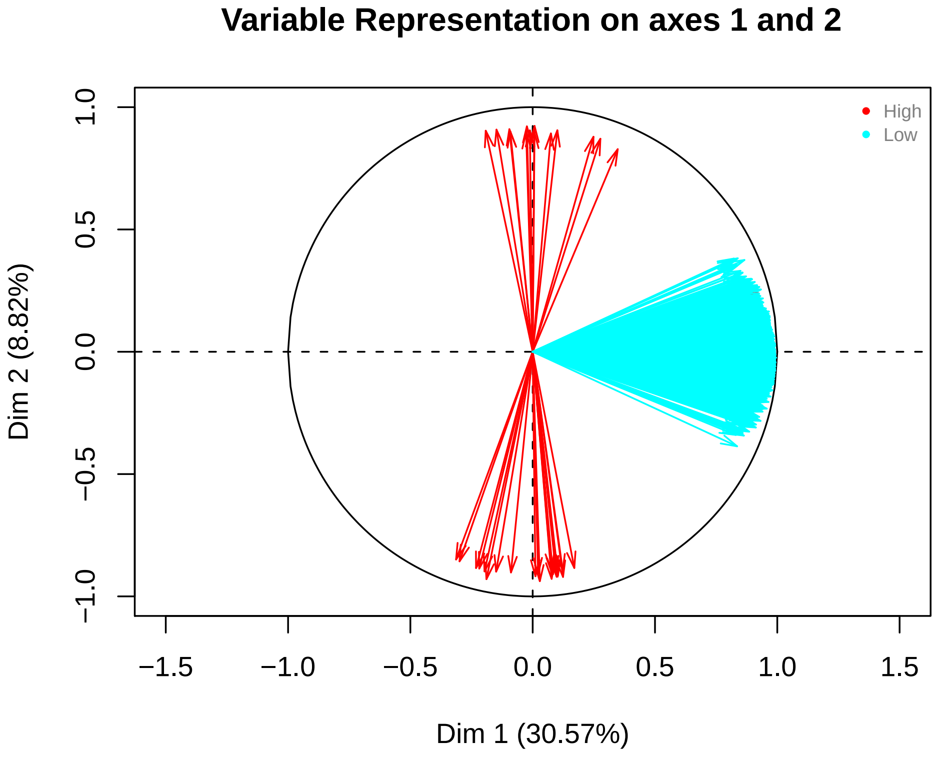

The main information provided by the data (summarised by axe 1) is not associated with the two groups of tumours. Only Axe 2 provides this information. It can be shown that Axe 1 is associated with the low expressed genes (high scaled signal level) rather than biological signal.

5.2 Agglomerative Hierarchical Clustering

As the PCA, the agglomerative hierarchical clustering is a useful method to identify clusters or to highlight experimental bias.
Clustering algorithm is based on the determination of two distances.
The first (called 'metric') defines the similarity between two elements. The second (called 'linkage criterion') define the similarity between two clusters of elements.
The choice of these distances is very important and depends on the similarities we want to detect (for example, the clustering of genes using the Pearson correlation or the Euclidean distance can lead to very different results). This kind of algorithm has shown to be sensitive to perturbations of the data. Thus, it is possible to assess the quality of the clustering method (based on a given metric and linkage criterion) by measuring its stability under perturbation or resampling of the data.

```
> ## Sample Hierarchical Clustering (Pearson's correlation coefficient and Ward method)
> c.sample<-clustering(data=marty.f, metric="pearson", method="ward")
> clustering.plot(tree=c.sample,  lab=marty.type.cl,
+                   title="GCRMA Data - filtered")
```

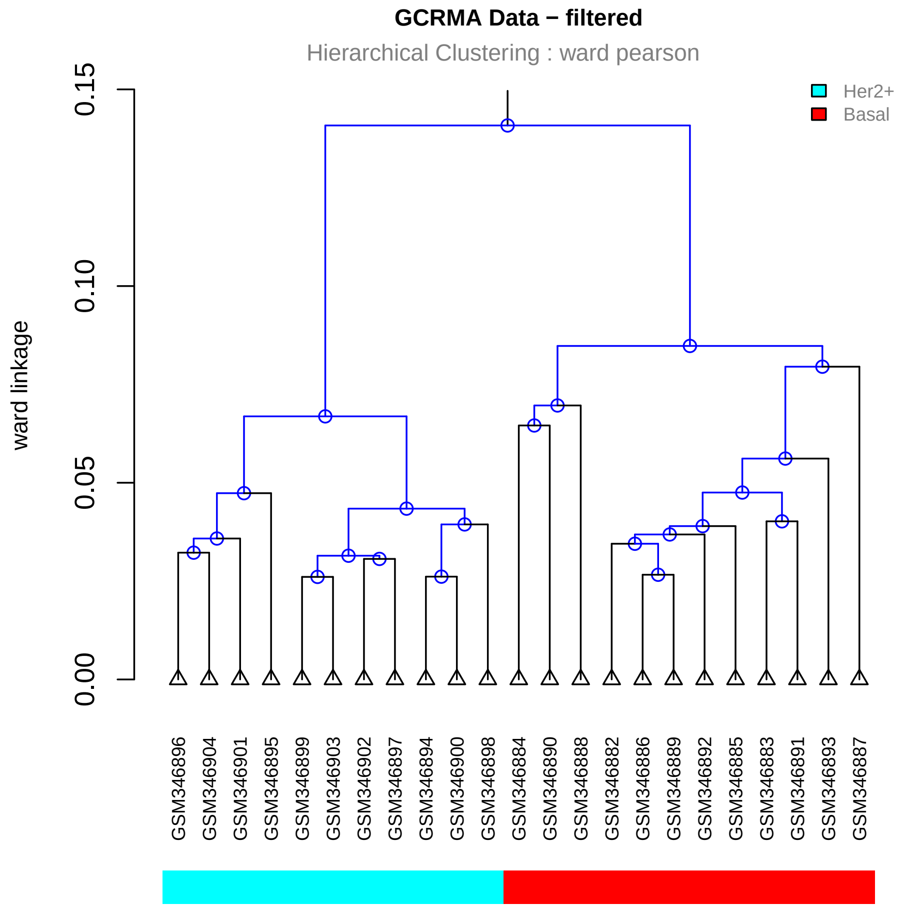

A heatmap is a graphical representation to visualise the level of expression of genes across the samples, and therefore allowing a better understanding of the clusters (genes and/or samples) detected. To facilitate the visualisation and the interpretation of the results, we propose to perform a gene selection (keep the genes with the highest interquartile range - IQR values) before performing the heatmap.

```
> ## Heatmap performed on the 100 probesets with the highest IQR values
> mvgenes<-genes.selection(marty.f, thres.num=100)
> c.sample<-clustering(data=marty.f[mvgenes,], metric="pearson", method="ward")
> c.gene<-clustering(data=t(marty.f[mvgenes,]), metric="pearsonabs", method="ward")
> clustering.plot(tree=c.sample, tree.sup=c.gene, data=marty.f[mvgenes,],
+                 names.sup=FALSE, lab= marty.type.cl, trim.heatmap=0.99)
```

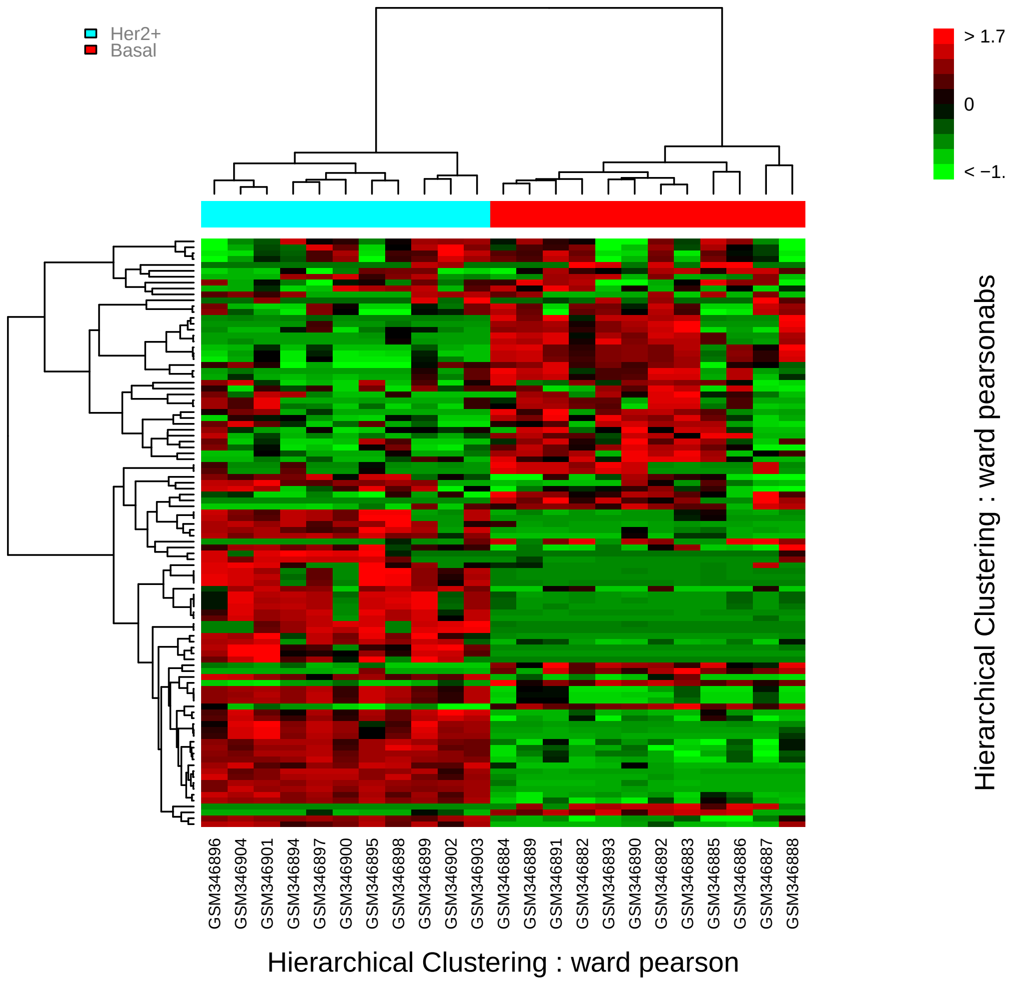

The two groups of tumours (HER2+ in red and BLCs in blue) are clearly separated by their gene expression profile, as also shown by the PCA.

6 Differential Analysis

A standard approach to detect Differentially Expressed Genes (DEG) is to test the difference between means of the two groups (with Student or Mann-Whitney tests for example) and then to adjust for multiple testing by applying the Benjamini and Hochberg (BH) procedure (Benjamini and Hochberg, 1995)

```
> ### Student test with BH correction and qqplot of genes.
> marty.type.num <- ifelse(marty.type.cl=="Her2+",0,1)
> rt<-runTtest(marty.f, labels=marty.type.num,algo="t.equalvar", q=0.05)
```

```
[1] "Launch  t.equalvar  test"
[1] "Calculate pval"
```

```
[1] "Adjusted pval"
[1] "typeFDR= FDR-BH"
[1] "3292 significant genes."
```

```
> head(rt)
```

```
   probeID        Stat  RawpValue AdjpValue
1   117_at  0.05985479 0.95283725 0.9758069
2   121_at  0.75142912 0.46073334 0.6481549
3   177_at  0.94302249 0.35639290 0.5661514
4 243_g_at -1.11306304 0.27826051 0.5100864
5 266_s_at -0.39507776 0.69676987 0.8213388
6   320_at  1.88783936 0.07294414 0.2481408
```

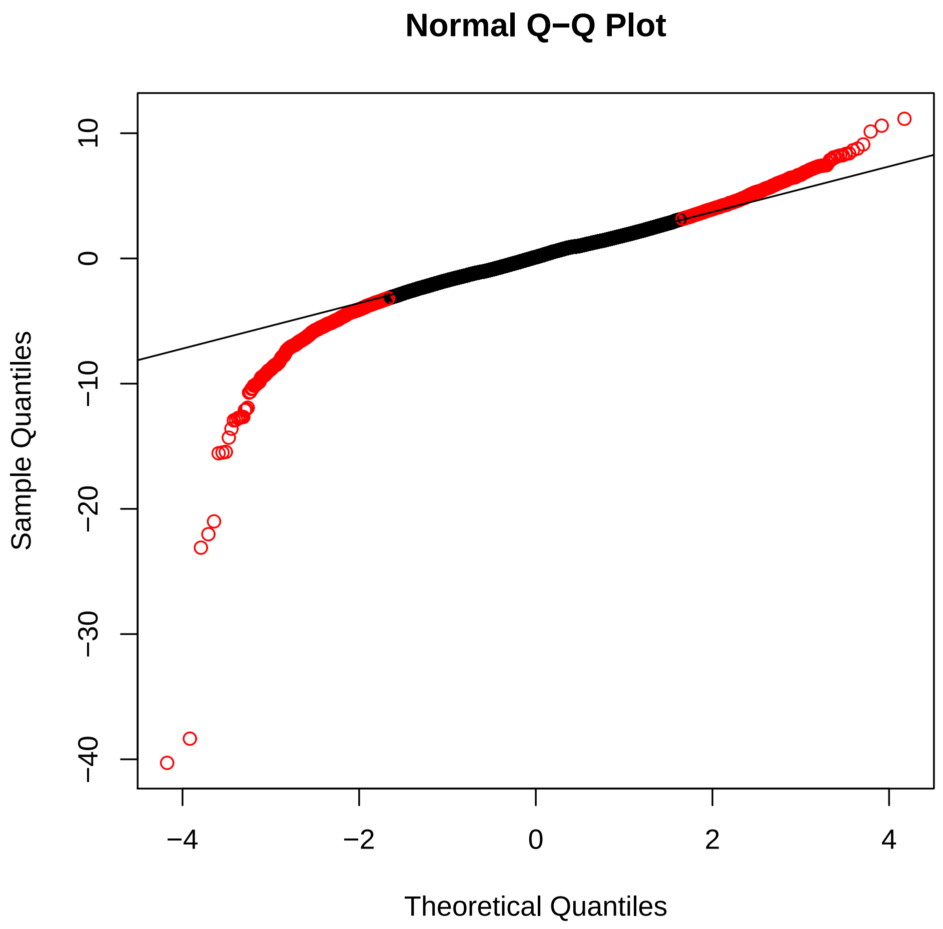

Detection of the DEG can also be performed by the Significance Analysis of Microarrays (SAM) algorithm (Tusher at al., 2001) (with t-test as statistic test for example). This algorithm is less stringent than the Benjamini and Hochberg procedure because it estimates the proportion of truly not differentially expressed genes.

By using permutations this approach takes into account the correlations between genes and avoids making assumptions about their distribution (which is usually problematic in microarray experiments due to a small sample size). This is an advantage over other techniques (for example t-test with Benjamini and Hochberg correction) which assume normal distribution with equal variance and independence of genes. In addition, test statistics of genes with low variability tend to be large,

which increases false positives. SAM corrects these statistics by adding a small value (called the 'fudge factor') to their denominator.
In case of more complex design experiments we also recommend the use of the limma package, very powerful tool to assess differential expression by using linear models.

```
> rs<-runSAM(marty.f, labels=marty.type.num)
> head(rs)
```

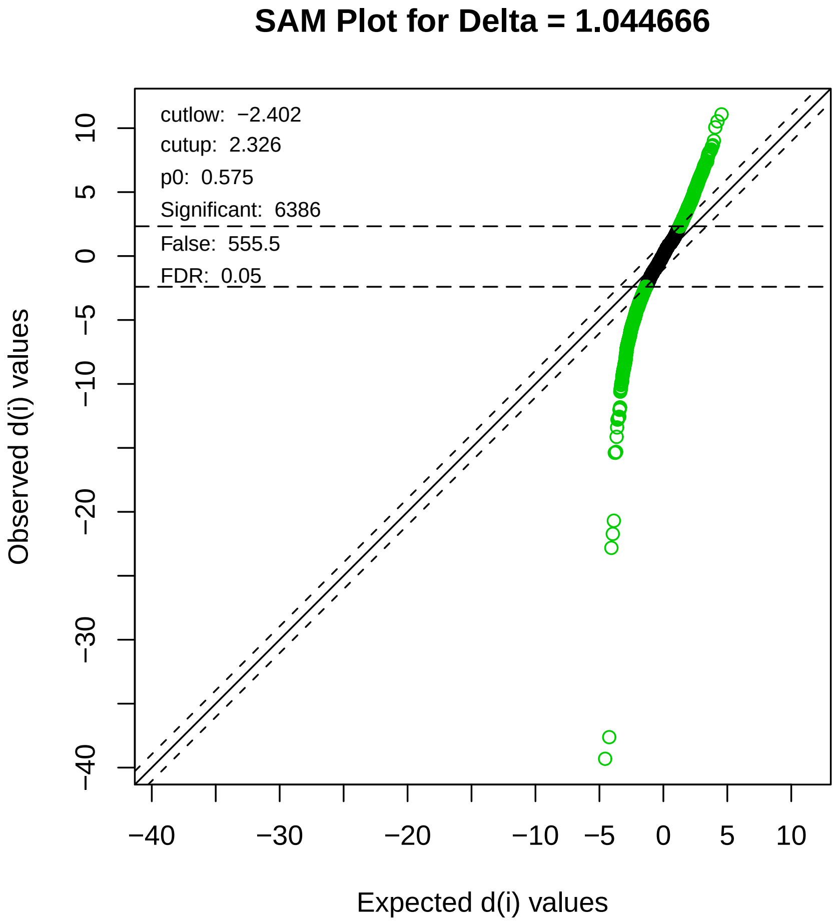

7 Gene annotation

Working with probesets name is often not the easiest way to interpret the data in term of biology. Annotations, as gene symbol are often very useful to understand the results. BioMart (http://www.biomart.org/) is a very powerful tool and gives direct access to several annotations databases (Ensembl, Uniprot, etc). A R package dedicated to interface R and the BioMart databases was created by Durinck S, Huber W, and Davis S.
EMA allows to query BioMart in a easiest way, without any knowledge of the biomaRt object. The connexion and the proxy configuration have to be checked before running biomaRt.

```
> ##Look at the annotation of the DEG
> rs.sorted <- rs[order(abs(rs$Stat), decreasing=TRUE),]
> rs.sign <- rs.sorted[which(rs.sorted[,"Significant"]),]
```

```
> rs.annot<-bioMartAnnot(rs.sign, inputTypeId ="affy_hg_u133_plus_2",
+                        outputTypeId=c("entrezgene","hgnc_symbol"),
+                        dataset=c("hsapiens_gene_ensembl"),database = "ensembl")

Checking attributes ... ok
Checking filters ... ok

> head(rs.annot)

     affy_hg_u133_plus_2     probeID      Stat RawpValue  FoldChange
2152           204667_at   204667_at -39.31048         0 0.017095646
9095           237086_at   237086_at -37.62050         0 0.007155071
2139           204623_at   204623_at -22.81831         0 0.009191687
2140           204623_at   204623_at -22.81831         0 0.009191687
8420         230323_s_at 230323_s_at -21.72791         0 0.017358886
7612           226226_at   226226_at -20.69646         0 0.028651862
     Significant entrezgene hgnc_symbol ensembl_gene_id
2152        TRUE         NA        <NA>            <NA>
9095        TRUE         NA        <NA>            <NA>
2139        TRUE         NA        TFF3 ENSG00000160180
2140        TRUE       7033        TFF3 ENSG00000160180
8420        TRUE     120224     TMEM45B ENSG00000151715
7612        TRUE     120224     TMEM45B ENSG00000151715
```

Annotations are an important issue of the microarray analysis. The probes used for the design of the array relied on earlier genome and transcriptome annotation and can be different from current knowledge. The first consequence of this problem is that many conclusions derived from analyses may be significantly flawed.
Here, all the analyses were done with the Affymetrix annotation package, but the users can also used their own probe set definition for their analysis.
EMA proposes a simple function to check the signal of the 11 probes summarized in each probeset.

```
> ## Not run because cel files are not available from this package
> filenames <- list.files("Data/E-GEOD-13787", pattern=".CEL", ignore.case=TRUE)
> rawdata <- ReadAffy(filenames=filenames, celfile.path="Data/E-GEOD-13787", cdfname=NULL)
> probePlots(rawdata, pbsList=rs.annot$affy_hg_u133_plus2[1:10])
```

8 Gene-Set analysis

Instead of working at the gene level, several recent studies discussed the problem of identifying differentially expressed groups of genes from a set of microarray experiments.
One of the most famous approach is the Gene Set Enrichment Analysis (GSEA) procedure of Subramanian et al. (Subramanian *et al.* (2006)). Here we decided to use the GSA package, presented in the paper as an improvement of the GSEA approach. It differs from a Gene Set Enrichment Analysis (Subramanian et al 2006) in its use of the ”maxmean” statistic: this is the mean of the positive or negative part of gene scores in the gene set, whichever is larger in absolute value. Moreover, GSA does ”restandardisation” of the genes, in addition of the permutation of columns, to estimate the false discovery rate.

```
> ## http://www.broad.mit.edu/gsea/msigdb/msigdb_index.html
> ## You have to register first and then download the gmt file from their site
> gsaOUT <- runGSA(marty, marty.type.num ,
+         gmtfile="c2.kegg.v2.5.symbols.gmt",
+         chip="hgu133plus2")
```

9 Functional enrichment assessment

The goal is to interpret the results provided by the differential analysis. One of the most classical way to answer this question, is to assess if the DEG list is enriched for biologically relevant gene-sets compared to a reference gene-set (called 'universe', typically all the genes of the array). Functional enrichment is based on the GeneOntology and KEGG pathways annotation terms.
The hyper-geometric test of the GOstats package (Falcon *et al.* (2007)) is used to test the over-representation of the functional terms in the gene list.
The choice of the universe could have a significant impact on the results. It is well discussed in the vignette of the GOstats package. Here, we decided to apply a non-specific filtering procedure different from the one proposed by Falcon and Gentleman. Since not all genes will be expressed under all conditions in our data, we can ask the question of defining the universe only with the expressed genes or with all the genes of the array. Actually, we are not able to distinguish the genes which are biologically non expressed, from the ones of low quality. That's why we think that the non-expressed probesets could be biologically relevant, as well as the ones with a little variation across samples, and we decided to first defined the universe with all the genes of the array. Then, we just remove probe sets that have no Entrez Gene identifier in our annotation data or no GO annotation. Finally, the Hypergeometric test is performed on the unique EntrezId of the gene list, and the unique EntrezId of the universe. The pvalues in output are not corrected from multiple testing. Note that because of the existing dependence structure (between genes, and GO terms) it is difficult to do any multiple testing correction. Moreover the most interesting genesets are not necessarily the ones with the smallest pvalues. Nodes that are interesting are typically those with a reasonable number of genes (10 or more) and small pvalues.
Another interesting method is provided by the globaltest package. It tests the null hypothesis that no gene in the gene set is associated with the class label rather than the null hypothesis that the genes in the gene set are not more associated with the class label than random genes on the microarray (followed by GSEA or methods based on hyper-geometric test). The model used for gene set testing is a regression model with random effects. It allows groups of genes of different size to be tested (from a single gene to all genes of the chip).

```
> ## GO and KEGG analysis on the DEG by the SAM procedure
> runHyperGO(list=rownames(rs.sign), pack.annot="hgu133plus2.db", name="HyperGO_type")
> runHyperKEGG(list=rownames(rs.sign), pack.annot="hgu133plus2.db", name="HyperKEGG_type")
```

Two types of reports are generated. The first one is an HTML report (see GOStats package) which describes the significant genesets (Id, pvalue, Odd Ratio, Expected Count, Count, Size and GO/KEGG term) The second (text format) describes the description of the content of each significant genesets, with the associated genes.

10 Supervised Classification

The classification is a prediction or learning problem from which each object is characterised by a response variable (class label) and a set of measurements (genes expression). One of the main goal of the supervised classification, especially in microarray experiments, is to find the best rule to

classify/distinguish the objects.
One of the most famous application of this type of method is the gene signature published by Van't veer as a predictor for metastasis (Van't Veer *et al.* (2002)). For all these approaches, we suggest to use the CMA package (Slawski *et al* (2008)) including the most popular machine learning and gene selection algorithms.

11 Survival analysis

Kaplan Meier and log-rank test analyses proposed by the EMA package helps for example to compare survival between patients with low expression value and patients with high expression value of a given gene.

```
> set.seed(5000)
> gene<-rnorm(100)
> gene[51:100]<-gene[51:100]+2
> group<-ifelse(gene<=median(gene),"Low gene expression","High gene expression")
> time<-abs(rnorm(100))
> time[51:100]<-time[51:100]+2
> status<-sample(c(0,1),size=100,replace=TRUE)
> res<-km(time,status,group,title="Kaplan Meier curve")
> res$fit.km

Call: survfit(formula = Surv(time, status) ~ group, na.action = na.omit)

                           records n.max n.start events median 0.95LCL 0.95UCL
group=High gene expression      50    50      50     23   2.85   2.643      NA
group=Low gene expression       50    50      50     27   1.34   0.845      NA

> res$lr

Call:
survdiff(formula = Surv(time, status) ~ group, na.action = na.omit)

                            N Observed Expected (O-E)^2/E (O-E)^2/V
group=High gene expression 50       23     33.4      3.22      10.2
group=Low gene expression  50       27     16.6      6.45      10.2

 Chisq= 10.2  on 1 degrees of freedom, p= 0.00142

> res$p.lr

[1] 0.001424340
```

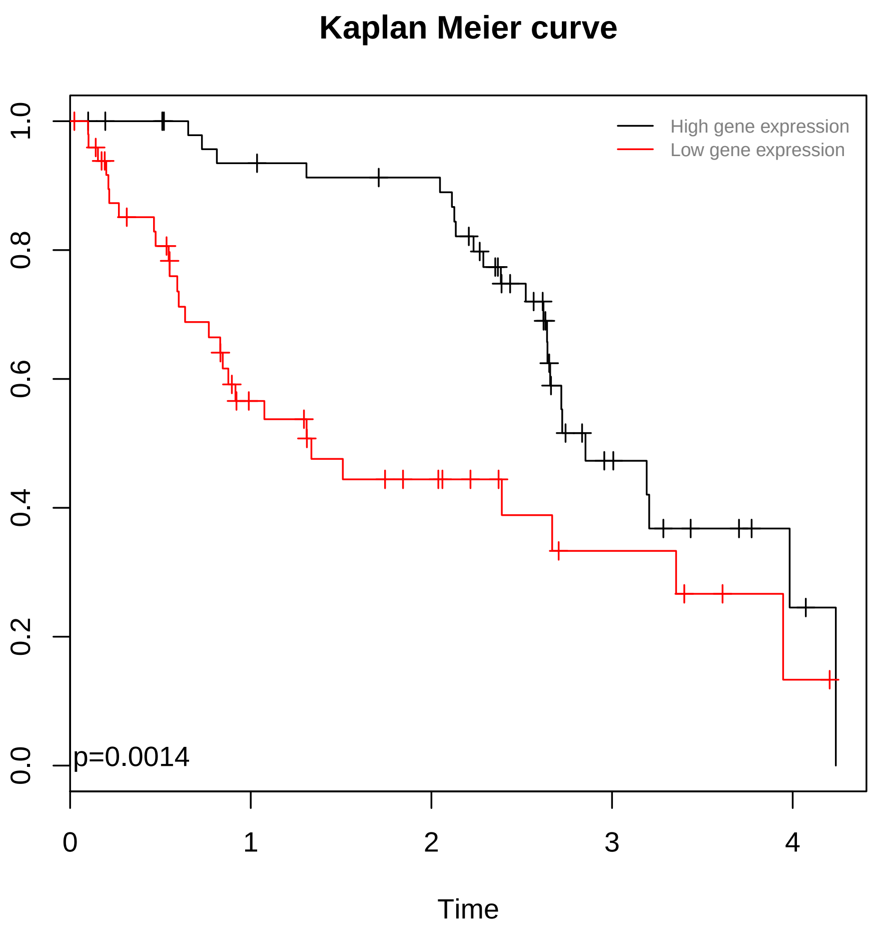

12 System Performance

We tested the EMA package on a classical desktop (1 cpu Intel Xeon 3.16 Ghz) and the performances were reported using the profr package.
The following table details how much CPU time each function used for the analysis workflow described above.

**function**	**time(s)**
data	0.24
expFilter	0.9
runPCA	7.4
plotSample	0.04
runPCA	8.58
plotVariable	11.06
clustering	0.1
clustering.plot	0.2
genes.selection	8.46
clustering	0.02
clustering.plot	0.42
runTtest	4.58
runSAM	77.12
bioMartAnnot	1.06
runHyperGO	186.86
runHyperKEGG	14.1
km	0.08

We also compared the performances of the GCRMA and RMA functions on a dataset of 290 HG-U133a2 Affymetrix arrays.
The normalisation was run on 1 CPU (3 Ghz) with a 64 bits version of R.2.11.1.

function	time(s)
RMA	350.961
GCRMA	481.055

As expected, GCRMA is more time computationally intensive than RMA on a large dataset. In this case, RMA can be an interesting alternative.

13 Session Info

- R version 2.10.0 (2009-10-26), `x86_64-unknown-linux-gnu`
- Locale: `LC_CTYPE=fr_FR.UTF-8`, `LC_NUMERIC=C`, `LC_TIME=fr_FR.UTF-8`, `LC_COLLATE=fr_FR.UTF-8`, `LC_MONETARY=C`, `LC_MESSAGES=fr_FR.UTF-8`, `LC_PAPER=fr_FR.UTF-8`, `LC_NAME=C`, `LC_ADDRESS=C`, `LC_TELEPHONE=C`, `LC_MEASUREMENT=fr_FR.UTF-8`, `LC_IDENTIFICATION=C`
- Base packages: base, datasets, graphics, grDevices, methods, splines, stats, utils
- Other packages: affy 1.24.2, AnnotationDbi 1.8.1, Biobase 2.6.1, biomaRt 2.2.0, Category 2.12.0, cluster 1.12.1, DBI 0.2-4, ellipse 0.3-5, EMA 1.2, FactoMineR 1.12, gcrma 2.18.0, GOstats 2.12.0, graph 1.24.1, GSA 1.0, heatmap.plus 1.3, Hmisc 3.7-0, lattice 0.17-26, MASS 7.3-4, multtest 2.2.0, RankProd 2.18.0, rgl 0.87, RSQLite 0.7-3, scatterplot3d 0.3-29, siggenes 1.20.0, survival 2.35-7, xtable 1.5-6
- Loaded via a namespace (and not attached): affyio 1.14.0, annotate 1.24.0, Biostrings 2.14.8, genefilter 1.28.2, GO.db 2.3.5, grid 2.10.0, GSEABase 1.8.0, IRanges 1.4.9, preprocessCore 1.8.0, RBGL 1.22.0, RCurl 1.3-0, tools 2.10.0, XML 2.6-0

References

Affymetrix Inc., (2002) Statistical Algorithms Description Document. *http://www.affymetrix.com/support/technical/whitepapers.affx*

Alston M.J., Seers J., Hinton J.CD., Lucchini S., BABAR: an R package to simplify the normalisation of common reference design microarray-based transcriptomic datasets, *BMC Bioinformatics* 2010, 11:73

Dai M., Wang P., Boyd A.D., Kostov G., Athey B., Jones E.G., Bunney W.E., Myers R.M., Speed T.P., Akil H., Watson S.J., Meng F., Evolving gene/transcript definitions significantly alter the interpretation of GeneChip data, *Nucleic Acids Research*, 33(20):e175.

Du P., Kibbe W.A., Lin S.M., *lumi*:a pipeline for processing Illumina microarray, *Bioinformatics.* 2008;24(13):1547-1548

Falcon S., Gentleman R, Using GOstats to test gene lists for GO term association, *Bioinformatics.* 2007 Jan 15;23(2):257-8

Huber W., Von Heydebreck A., SÃ¼ltmann H., Poustka A. and Vingron M. (2002) Variance stabilization applied to microarray data calibration and to the quantification of differential expression.*Bioinformatics* 18: suppl. 1 (2002), S96-S104 (ISMB 2002).

Gentleman, R. C. and Carey, V. J. and Bates, D. M. and Bolstad, B. and Dettling, M. and Dudoit, S. and Ellis, B. and Gautier, L. and Ge, Y. and Gentry, J. and Hornik, K. and Hothorn, T. and Huber, W. and Iacus, S. and Irizarry, R. and Leisch, F. and Li, C. and Maechler, M. and Rossini, A. J. and Sawitzki, G. and Smith, C. and Smyth, G. and Tierney, L. and Yang, J. Y. and Zhang, J., Bioconductor: open software development for computational biology and bioinformatics, *Genome Biology*, 2004, 5, R80.

Irizarry R.A., Hobbs,B. et al. (2003) Exploration, normalisation, and summaries of high density oligonucleotide array probe level data, *Biostatistics*, **4**, 249-254

Kauflmann,A., Gentleman R., Huber W. (2009) ArrayQualityMetrics-a bioconductor package for quality assessment of microarray data, *Bioinformatics*,vol. 25, no3.pp. 415-416

Marty, B., et al. (2008) Frequent PTEN genomic alterations and activated phosphatidylinositol 3-kinase pathway in basal-like breast cancer cells,*Breast Cancer Res.*, 10, R101.

Slawski M., Daumer M., Boulesteix A.L., CMA - A comprehensive Bioconductor package for supervised classification with high dimensional data, *BMC Bioinformatics*, Vol. 9, No. 1.

Subramanian A., et al. Gene set enrichment analysis: A knowledge-based approach for interpreting genome-wide expression profiles, *Proc. Natl. Acad. Sci. USA*, 102, 15545-15550.

Smyth, G. K., and Speed, T. P. (2003). Normalization of cDNA microarray data. Methods 31, 265-273.

Van 't Veer,L.J., et al. (2002) Gene expression profiling predicts clinical outcome of breast cancer, *Nature*, 415, 530-536.

Wu,Z., Irizarry R.A., et al. (2004) A Model-Based Background Adjustment for Oligonucleotide Expression Arrays, *Journal of the American Statistical Association*, 99, 909.
